# Effects of Bonded Rapid Palatal Expander on Vertical Dimension: A Systematic Review and Meta-Analysis

**DOI:** 10.3390/jcm14197035

**Published:** 2025-10-04

**Authors:** Sarah Horne, Doyeon Sung, Hugo Cesar Campos, Shahd Habeb, Luca Sfogliano, Chun-Hsi Chung, Chenshuang Li

**Affiliations:** 1 Department of Orthodontics, School of Dental Medicine, University of Pennsylvania, Philadelphia, PA 19104, USA; 2 School of Dental Medicine, University of Pennsylvania, Philadelphia, PA 19104, USA; 3 Department of Diagnostic Sciences, Tufts University School of Dental Medicine, Boston, MA 02111, USA; 4 Department of Graduate Orthodontics, University of Detroit Mercy, Detroit, MI 48208, USA

**Keywords:** bonded rapid palatal expander, rapid maxillary expansion, vertical dimension

## Abstract

**Objectives:** The current study aimed to summarize the current evidence on vertical control provided by the bonded rapid palatal expander (BRPE) in pediatric patient populations within 6 months after expansion. **Methods:** Relevant studies were screened independently by two researchers from the eight databases MEDLINE (PubMed), Web of Science, SCOPUS, Embase, Cochrane, LILACS (Latin American and Caribbean Health Sciences Literature), LIVIVO and Google Scholar, and supplemented by a manual search of the reference lists from studies selected for full-manuscript reading. Relevant data from lateral cephalograms taken pre- and post-expansion was extracted. A meta-analysis was performed with RStudio and a risk of bias assessment of the included articles was completed. **Results:** Ten relevant studies were included for data extraction, although most had a high risk of bias. The meta-analysis revealed that within 6-month retention after BRPE treatment, there were (1) slight increases in total (0.83 mm), upper (0.57 mm), and lower (0.70 mm) facial height; (2) minimum change in the palatal plane angulation (−0.01°); (3) minimum change in the occlusal angulation (−0.04°); and (4) minimal mandibular plane angulation changes with 0.01° increase in SN-GoGn angle, 0.71° increase in SN-MP angle, 0.17° increase in FMA, and 0.82° increase in PP-GoGn angle. **Conclusions:** Current evidence indicates that BRPEs may not control or reduce the vertical dimension significantly within 6 months after expansion. Further high-quality studies, particularly on hyperdivergent patients, are needed to clarify whether bonded expanders offer advantages over traditional banded expanders in management of the vertical dimension.

## 1. Introduction

Orthodontic treatment frequently requires the correction or management of the three dimensions of occlusion: sagittal, vertical, and transverse. The vertical component is particularly important to manage in patients presenting with high angle growth patterns or tendencies toward open bite malocclusions [1]. Uncontrolled vertical growth can compromise both functional outcomes and esthetics, making vertical control a key consideration when planning treatment for a case [2].

Rapid palatal expansion (RPE) has long been used to correct maxillary transverse deficiencies. Traditional banded expanders, while effective in achieving skeletal expansion, have been associated with undesirable skeletal side effects such as downward displacement of the maxilla, downward and backward movement of the mandible, and an overall increase in lower facial height [3]. Lagravere et al. found in a systematic review that in patients treated with rapid maxillary expansion, the maxillary molar cusp extruded 0.53 mm in relation to the palatal plane, although this change is not clinically significant [4]. Nonetheless, these skeletal and dental changes can be problematic in hyperdivergent patients or those predisposed to vertical excess.

To address the issue mentioned above, bonded rapid palatal expanders (BRPEs) have gained popularity among clinicians as a modified approach to traditional RPEs. BRPEs are typically designed with acrylic coverage over the occlusal surfaces of posterior teeth, functioning similarly to bite blocks. Theoretically, this design feature infringes on the patient’s Freeway Space, resulting in a stretch of the elevator muscles beyond their normal resting length. As a result, an intrusive force is thus directed towards the maxillary and mandibular teeth from the tension in the elevator facial muscles [5]. This theory is echoed by some research, which demonstrated that bonded expanders decreased the amount of inferior movement of the maxilla during the expansion when comparing to banded RPE [5,6]. However, the evidence remains varied, with some studies reporting minimal vertical controlling effects from BRPE [7,8].

Given the importance of vertical control in achieving optimal orthodontic outcomes—particularly in patients with vertical growth tendencies, the evaluation of bonded expanders as a tool for managing the vertical dimension merits detailed investigation. This systematic review and meta-analysis aim to summarize and analyze the currently available information regarding the effect of the BRPE on the skeletal vertical dimension, as well as molar vertical position, within 6 months after expansion without the interference of other orthodontic appliances.

## 2. Materials and Methods

The current systematic review protocol is registered on Open Science Framework Registries (osf.io/u4r2j). All original articles were accessed through a thorough database search from the following electronic databases: MEDLINE (PubMed), Web of Science, SCOPUS, Embase, Cochrane, LILACS (Latin American and Caribbean Health Sciences Literature), ZBMed (LIVIVO) and Google Scholar, with an initial search finish date of 25 November 2024.

### 2.1. Study Selection Criteria

**Figure 1:**
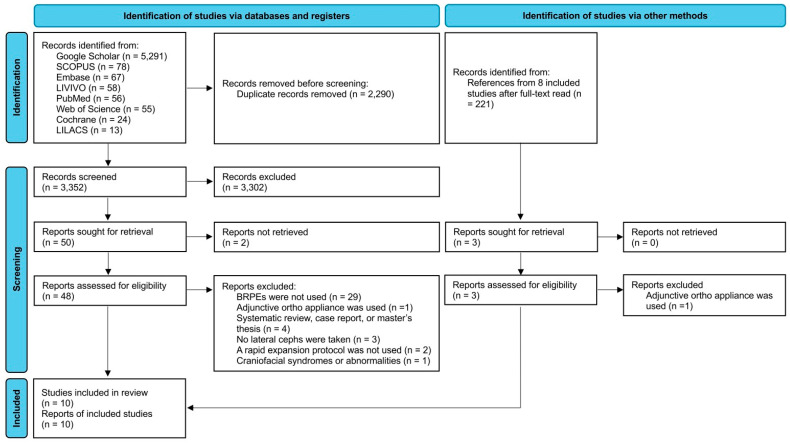
The PRISMA flow diagram demonstration the study identification and screening.

Using the population, interventions, comparison, and outcomes (PICOs) framework (Table 1), we conducted a systematic literature review on the effect of BRPEs on the vertical dimension. The inclusion criteria comprised (1) longitudinal studies (both prospective and retrospective) comparing pre- and post-expansion records, (2) use of lateral cephalometric radiographs to measure vertical parameters, and (3) use of an expander with posterior occlusal acrylic coverage to achieve expansion. The exclusion criteria were (1) BRPEs were not used or an alternative expander was used, such as a quad helix expander; (2) the study protocol included use of adjunctive orthodontic appliances other than BRPEs that could influence vertical parameters, such as a chin cup, facemask, or temporary anchorage devices (TADs), or orthodontic intervention was performed with the mandibular arch in conjunction with the maxillary expansion; (3) the article was a systematic review, case report, opinion, conference abstract, editorial, or master’s thesis; (4) the expansion protocol did not adhere to the rapid protocol; and (5) patients with craniofacial syndromes or other abnormalities were included in the study. No language or date restrictions were imposed. The PRISMA flow diagram illustrates the process of obtaining the final included articles (Figure 1).

### 2.2. Search Strategy

Our search strategy in all included databases was as follows: (“maxillary expander” AND “vertical”), (“maxillary expander” AND “intrusion”), (“Haas expander” AND “vertical”), (“Haas expander” AND “intrusion”), (“palatal expander” AND “vertical”), (“palatal expander” AND “intrusion”), (“Hyrax expander” AND “vertical”), and (“Hyrax expander” AND “intrusion”). We also performed a manual review of the references cited in the articles that we identified for full-text reading. The full texts of these articles were thoroughly evaluated and compared to the predetermined inclusion and exclusion criteria. Two authors (S. Horne and D.S.) independently carried out the literature search and screening to ensure the reliability and comprehensiveness of the results. In cases of discrepancies between the two authors, a third author (C.L.) was consulted for further discussion. All non-English articles were evaluated with the assistance of authors who were proficient in the written language (S. Habeb, H.C.C., and L.S.).

### 2.3. Risk of Bias/Quality Assessment

To assess the risk of bias, we modeled our protocol after that of a similar systematic review by Shen et al. [9]. Risk of bias was evaluated across four categories: study design, study measurements, statistical analysis, and other factors (Table 2). Two authors (S.H. and D.S.) independently assessed each article, and any discrepancies were resolved by a third author (C.L.). The articles [10,11] written in non-English language, Hasan et al. and Mara Galon et al., were assessed by authors proficient in the written language (S. Habeb and H.C.C., respectively). A final score for each article was calculated by dividing the number of criteria met by the total number of criteria (17). Based on this score, articles were classified as having a high, medium, or low risk of bias. An article was rated as high-risk if it did not meet or did not address ten or more of the relevant criteria. A low risk of bias was assigned when all criteria were clearly met. Articles that partially met the criteria or where the information was unclear were classified as medium-risk.

### 2.4. Data Extraction and Analysis

For all the articles included for further data analysis, relevant information was extracted from each article, including study type, sample size, gender, age range of patients, type and design of expander used, type of records, and timing of treatment records (Table 3), as well as the parameters used to evaluate skeletal changes in the vertical dimension and the vertical position of permanent first molars (Table 4).

After completing the systematic review and data collection process, we observed there was inconsistency in the timing of post-treatment imaging, with some studies using lateral cephalograms taken immediately after expansion (T2), while others used those taken after a retention period of up to six months (T3). After reviewing all the available data, meta-analysis was performed on any parameter that was measured by two or more studies and with the quantitative change between time points for these parameters in the format of mean ± standard deviation that either was directly stated in the study or could be calculated based on the report of the T1 and T3 values.

### 2.5. Statistical Analysis

The outcomes of this study that were analyzed included changes in (1) total, upper, and lower facial height, (2) palatal plane angulation and position, (3) vertical position of the upper and lower first molars as well as the occlusal plan angulation, and (4) overall skeletal measurements related to the mandibular lower border to assess the vertical dimension. The meta-analysis was performed on the extracted data using RStudio (version 2023.09.1+494, Posit Software, PBC, Boston, Massachusetts, USA) [16,17]. The meta-analysis was carried out employing a random effects model, and heterogeneity was evaluated for variance among studies using the Tau^2^ method (τ^2^). Results were presented as a Mean and 95% confidence interval [CI]. Sensitivity analysis and selective reporting within studies were not evaluated due to the limited number of studies included per analyzed variable.

## 3. Results

### 3.1. Literature Searching and Study Selections

Through the initial search of the seven databases, 5642 potentially relevant articles were identified (Figure 1). Following removal of duplicate articles, a total of 3352 articles remained for title screening and abstract review to remove irrelevant articles. Following title and abstract screening, 50 articles remained for full-text retrieval. Two articles could not be retrieved, and three additional articles were retrieved following reference list reviews. Finally, ten total articles met the inclusive and exclusive criteria and remained for the systematic review and analysis [5,6,7,8,10,11,12,13,14,15].

### 3.2. Risk of Bias

The quality of evidence from the ten studies included in our systematic review was evaluated using a risk of bias assessment (Table 2). Only Asanza et al. reported random allocation of treatment [6], while four studies did not perform randomization [12,13,14,15]. For the remaining studies, it was unclear whether randomization was performed [5,7,8,10,11]. None of the studies reported blinding of the examiner or statistician. Intra-rater reliability was assessed in five studies [7,10,12,13,14], and inter-rater reliability was assessed in one study [13]. Based on our analysis, none of the studies were classified as having a low risk of bias. Five studies were determined to have a medium risk of bias [7,10,12,13,14], and five were assessed as having a high risk of bias [5,6,8,11,15].

### 3.3. Demographic Data

The characteristics of the included study are shown in Table 3. Of the ten included studies, three were prospective, one was retrospective, and six did not clearly state whether they were prospective or retrospective studies. The expansion protocol for the studies was similar, with most using a typical rapid expansion protocol of two turns per day. All studies included lateral cephalometric radiographs taken before and after treatment, although the point at which the post-treatment records were taken ranged from immediately following expansion to up to 6 months retention.

Each study measured different vertical parameters, which is demonstrated in Table 4. Since a limited number of studies reported the measurements immediately after expansion (T2, i.e., within one month since treatment started), the meta-analysis was performed on the changes between T1 and T3 time points to show the effects of BRPEs after short-term retention.

### 3.4. Facial Height

**Figure 2:**
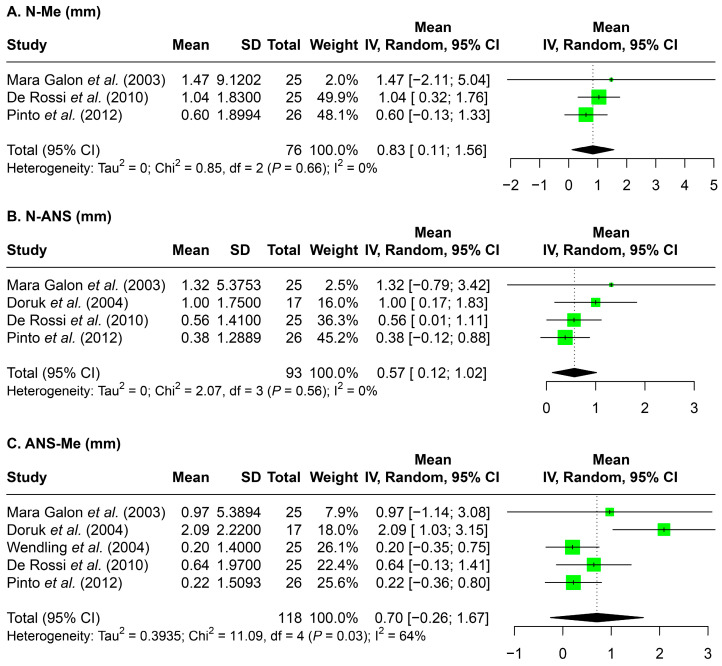
Forest plots detailing the meta-analysis results for facial height: (**A**) N-Me, (**B**) N-ANS, and (**C**) ANS-Me. SD: standard deviation; CI: confidence interval; N: nasion; Me: menton; ANS: anterior nasal spine; mm: millimeters. Citation of involved references: [7,8,11,14,15].

Changes in upper, lower, and total facial height were presented in Figure 2. Overall, a slight increase in facial height was evident for all three measurements. The greatest increase was seen in total facial height (N-Me, 0.83 mm), followed by upper facial height (N-ANS, 0.07 mm) and lower facial height (ANS-Me, 0.57 mm).

### 3.5. Palatal Plane Angulation and Position

**Figure 3:**
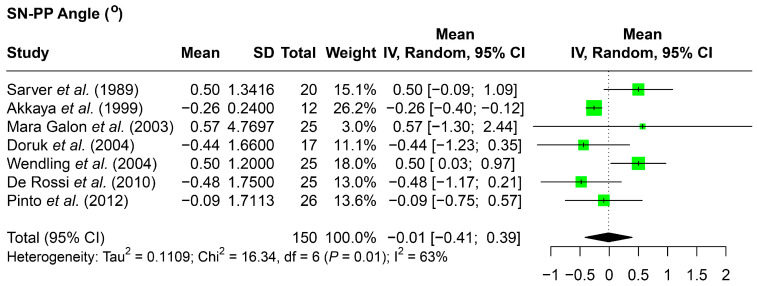
Forest plots detailing the meta-analysis results for the skeletal vertical parameters evaluating palatal plane angulation as SN-PP angle. SD: standard deviation; CI: confidence interval; SN: sella nasion line; PP: palatal plane. Citation of involved references: [5,7,8,11,12,14,15].

Limited data was available to assess the palatal plane vertical position, with two studies reporting a greater increase in the SN-ANS distance observed in comparison to the SN-PNS distance (Table 4) [5,6]. However, the meta-analysis revealed a minimal change in palatal plane angulation when compared to the SN plane (SN-PP angle, −0.01 degrees) (Figure 3).

### 3.6. Molar Position and Occlusal Plane Angulation

To evaluate the effect of bonded expanders on the vertical position of the upper and lower first molars, the distance of the upper first molar to the palatal plane and the lower first molar to the mandibular plane was reported (Table 4), with two studies reporting a reduction in the distance between the upper first molar to the palatal plane [6,13], and one study reporting an increase in the distance between the lower first molar to the mandibular plane [13]. However, no sufficient data could be utilized for a meta-analysis.

**Figure 4:**
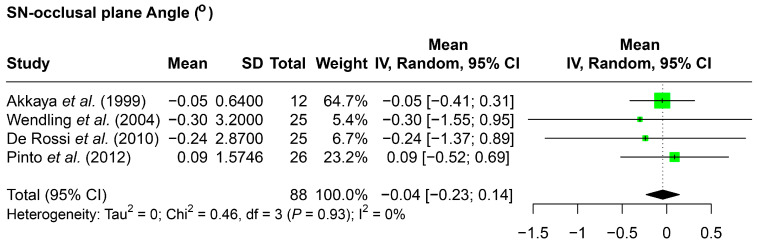
Forest plot detailing the meta-analysis results for the SN–occlusal plane angle. SD: standard deviation; CI: confidence interval; SN: sella nasion line. Citation of involved references: [7,8,12,15].

With the vertical position changes of the molars, the angle between the sella-nasion line and the occlusal plane (SN–occlusal plane) was also assessed, which demonstrated a little decrease (−0.04 degrees) (Figure 4).

### 3.7. Skeletal Assessments of the Vertical Dimension

**Figure 5:**
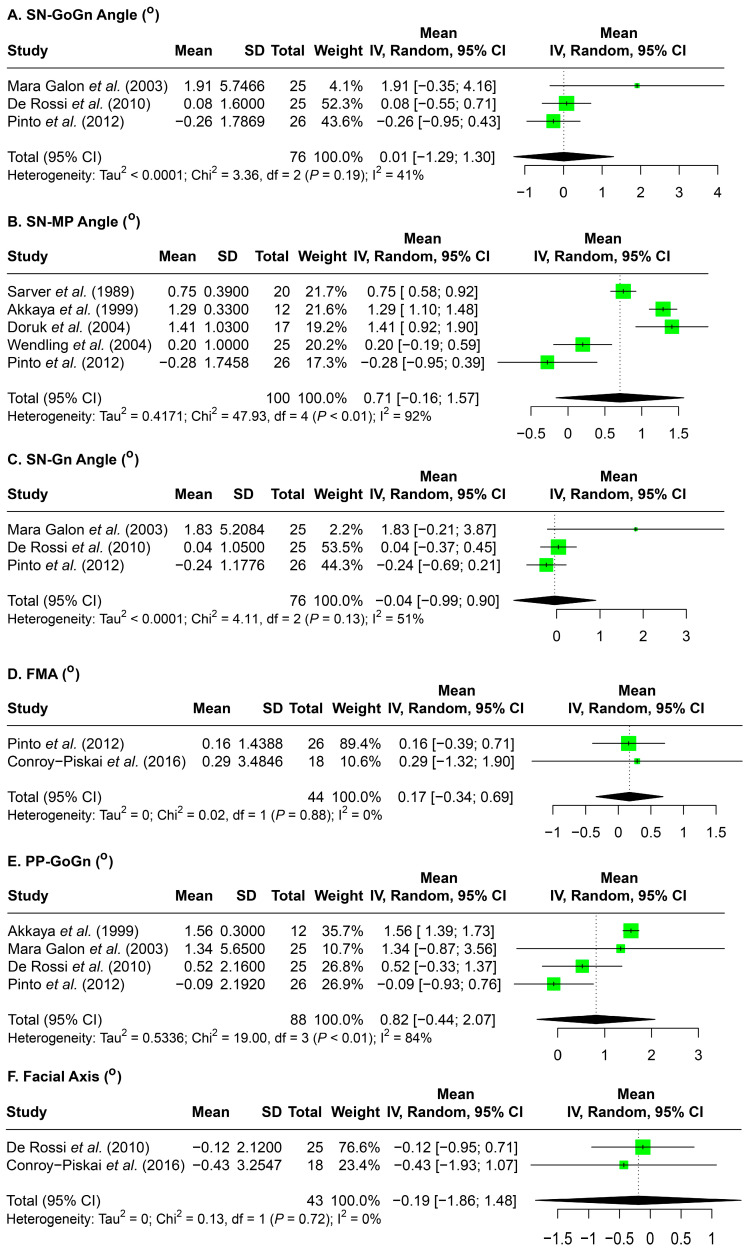
Forest plots detailing the results of the meta-analysis for overall skeletal measurements related to the mandibular lower border to assess the vertical dimension: (**A**) SN-GoGn angle, (**B**) SN-MP angle, (**C**) SN-Gn angle, (**D**) FMA, (**E**) PP-GoGn, and (**F**) facial axis. SD: standard deviation; CI: confidence interval; SN: sella nasion line; GoGn: gonion–gnathion line; MP: mandibular plane; Gn: gnathion; FMA: Frankfort–mandibular plane angle; PP: palatal plane. Citation of involved references: [5,7,8,11,12,13,14,15].

Lastly, the changes in the skeletal aspect of the vertical dimension in relation to the mandibular lower border were assessed (Figure 5). Overall, minimal changes were observed across these measurements. SN-Gn angle decreased by 0.04°, and facial axis by 0.19°. Increases were seen in SN-GoGn angle (0.01°), SN–mandibular plane angle (0.71°), FMA (0.17°) and PP-GoGn angle (0.82°).

## 4. Discussion

### 4.1. Summary of Evidence

The use of BRPEs with occlusal acrylic coverage, as opposed to banded RPEs, is a common orthodontic treatment approach aimed at minimizing increases in the vertical dimension during maxillary expansion. Despite its widespread use, no systematic review or meta-analysis to date has consolidated the evidence supporting this approach or clarified the mechanisms by which bonded expanders may control the vertical dimension. This systematic review and meta-analysis analyzed data from ten studies utilizing pre- and post-expansion lateral cephalometric radiographs to evaluate changes in the vertical dimension. Four outcomes were assessed: facial height, palatal plane position and angulation, vertical position of the upper and lower permanent first molars, and the vertical position of the mandibular lower border.

The literature search resulted in many studies with high variability in study characteristics; thus, only ten articles were included in our systematic review and nine articles were utilized in our meta-analysis. Overall, the mean changes in the skeletal parameters of the vertical dimension were small or insignificant within 6 months post-expansion. An important consideration when interpreting the results of the meta-analysis is that, as the involved subjects are children or teenagers, the observed mean changes in vertical parameters cannot be solely attributed to BRPE treatment. These changes may also reflect normal dentofacial growth. Buschang et al. reported that facial height increases by approximately 2.4 mm per year in males and 1.9 mm per year in females as part of typical growth patterns [18], while the current study found 0.83 mm of facial height increase after BRPEs intervention within 6 months post expansion. In terms of angular changes, an annual increase of 0.3 to 0.4 degrees in SN-MP and FMA is reported by Buschang et al. [18], and our current study demonstrated 0.71 degree and 0.17 degree changes, respectively. Therefore, bonded expanders likely do not result in an increase or decrease in the vertical dimension but may maintain the vertical dimension throughout treatment.

It is also important to consider if BRPEs provide a superior effect in vertical control when comparing to banded RPEs. A systematic review by Lione et al. that mixed both bonded and banded RPEs as well as mixed studies reporting short-term and long-term effects concluded that RPE resulted in a 1.7-degree increase in SN-MP angle and a 1.1-degree increase in SN-GoGn angle [19]. Another systematic review by Lagravere et al., which also combined banded and bonded Hyrax expanders, stated that immediately after expansion, there was a 1.97 increase in the SN-MP angle and a 1.65 degrees increase in the PP-MP angle [4]. The changes on the above parameters are about one degree larger than the result of our current study where a 0.71 degree increase in SN-MP, a 0.01 degree increase in SN-GoGn, and a 0.82 degree increase in PP-GoGn angle were revealed after bonded RPE treatment (Figure 5). Thus, the difference in the vertical effects of the banded and bonded RPE might be very minimal, with bonded expanders providing slightly more vertical control. In fact, Reed et al. compared patients treated with a banded versus a bonded RPE followed by edgewise orthodontics, and concluded that the banded RPE group had more vertical changes than the bonded RPE group, but most of the changes were less than 1 degree [20], which may not have clinical significance. However, it is worth noting that the initial vertical skeletal pattern of the involved subjects was not reported in these studies. Since vertical control is more critical for patients with a hyperdivergent pattern, further clinical studies that directly compare the short- and long-term effects of bonded and banded RPEs on patients with hyperdivergent pattern are needed. Such data will allow clinicians to individualize treatment planning based on both transverse and vertical treatment goals. A thorough understanding of these concepts will ultimately support orthodontists in delivering more predictable, efficient, and patient-specific care for individuals presenting with transverse maxillary deficiencies and varying vertical growth tendencies.

### 4.2. Limitations

There are several limitations to the current systematic review and meta-analysis that must be acknowledged. First, despite a comprehensive literature search, the number of studies meeting inclusion criteria was limited. The current body of published research specifically addressing the effects of BRPEs on the vertical dimension remains sparse. Consequently, our meta-analysis was based on a relatively small pool of studies and patients, which may reduce the statistical power and generalizability of our findings. Second, there was considerable heterogeneity among the included studies. This variability was reflected in the wide 95% confidence intervals observed for many of the mean changes in vertical measurements, making it challenging to draw definitive conclusions regarding the true effects of BRPEs on the vertical dimension. Furthermore, the outcomes evaluated in the meta-analysis represent mean changes between pre- and post-treatment lateral cephalometric tracings. These changes may reflect both treatment-related effects and natural growth and development. As such, it is not possible to isolate and attribute the observed skeletal or dentoalveolar changes solely to the use of bonded expanders. Future studies that include control groups or growth-matched comparisons will be critical in addressing this limitation.

## 5. Conclusions

The current research on bonded expanders and the treatment effects on the vertical dimension is limited and highly variable. Our systematic review and meta-analysis showed that bonded expanders may not control or reduce the vertical dimension significantly.

## Figures and Tables

**Table 1:** The PICO questions of this study.

Criteria	Description
Population	Growing patients with a maxillary transverse discrepancy
Intervention	Bonded rapid palatal expander
Comparisons	Pre-treatment records
Outcome	Change in skeletal and dentoalveolar vertical parameters

**Table 2:** Risk of bias assessment of the ten included studies. “+”: low risk of bias; “?”: medium risk of bias; “-”: high risk of bias.

		Akkaya et al. (1999) [12]	Asanza et al. (1997) [6]	Conroy-Piskai et al. (2016) [13]	De Rossi et al. (2010) [8]	Doruk et al. (2004) [14]	Hasan et al. (2019) [10]	Mara Galon et al. (2003) [11]	Pinto et al. (2012) [7]	Sarver et al. (1989) [5]	Wendling et al. (2005) [15]
Study Design	Objective: objective clearly formulated	+	+	+	+	+	+	-	+	+	+
	Sample size: considered adequate and estimated before collection of data	?	?	?	?	?	+	-	?	+	+
	Baseline characteristics: similar baseline characteristics	+	+	+	+	-	+	-	+	-	+
	Co-interventions (retainer, etc.)	+	+	+	+	-	-	-	+	+	+
	Randomization										
	Random sampling	-	-	-	?	-	?	?	?	?	-
	Random allocation of treatment	-	+	-	?	-	?	?	?	?	-
Study Measurements	Measurement method: appropriate to the objective	+	+	+	+	+	+	-	+	+	+
	Blind measurement: blinding										
	Blinding (examiner)	-	-	-	-	-	?	-	-	-	-
	Blinding (statistician)	-	-	-	-	-	?	-	-	-	-
	Reliability										
	Reliability described? (intra-rater reliability)	+	-	+	-	+	+	?	+	-	-
	Adequate level of agreement? (inter-rater reliability)	-	-	+	?	-	?	?	-	-	-
Statistical Analysis	Statistical analysis										
	Appropriate for data	?	+	+	?	?	+	-	?	?	?
	Combined subgroup analysis	?	?	?	?	?	+	-	?	?	?
	Cofounders (co-interventions): confounders included in analysis	-	-	-	-	-	?	-	-	-	-
	Statistical significance level										
	*p*-value stated?	+	+	+	+	+	+	-	+	+	+
	Confidence intervals stated?	-	-	+	-	-	+	?	-	-	-
Other	Clinical significance	+	+	+	+	+	+	-	+	+	+
Total score		7	8	10	6	5	10	0	7	6	7
Percentage of the total		41.18	47.06	58.82	35.29	29.41	58.82	0	41.18	35.29	41.18
Risk of bias		MED	HIGH	MED	HIGH	MED	MED	HIGH	MED	HIGH	HIGH

**Table 3:** Characteristics of the ten included studies. F: female; M: male.

Study	Study Type	Mean Patient Age	Patient Age Range	Sample Size (F/M)	Expansion Protocol	Treatment Time	Time of Post-Expansion Records
Akkaya et al. (1999) [12]	Unclear	11.96 years	10.40–13.50 years	12 (5/7)	2 turns/day	0.70–1.60 months	Immediately post expansion and at 3 months retention
Asanza et al. (1997) [6]	Unclear	Not specified	Not specified	Not specified	2 turns/day	Not specified	Immediately post expansion and at 3 months retention
Conroy-Piskai et al. (2016) [13]	Retrospective	102.89 ± 8.90 months	Not specified	18 (14/4)	2 turns/day	12.28 ± 8.29 months	At 6 months retention
De Rossi et al. (2010) [8]	Unclear	8 years 5 months	6 years 11 months–11 years 5 months	25 (13/12)	1/2 turn/day	20 days (14–26 days)	At mean of 107 days (90–124 days)
Doruk et al. (2004) [14]	Unclear	12.7 ±1.1 years	Not specified	17 (10/7)	1/2 turn/day	26.47 ± 2.85 days	At 90 days retention (90.4 ± 6.7 days)
Hasan et al. (2019) [10]	Prospective	-	6–12 years	18 (F/M not specified)	1 turn/day	Not specified	At 6 months retention
Mara Galon et al. (2003) [11]	Prospective	-	7.4–14.1 years	25 (13/12)	3 turns/day	Not specified	At 4 months retention
Pinto et al. (2012) [7]	Unclear	8 years 5 months	6 years 11 months–10 years 11 months	26 (15/11)	2 turns/day	Not specified	At mean of 107 days (90–124 days)
Sarver et al. (1989) [5]	Unclear	10.8 years	7.5–16 years	20 (14/6)	2 turns/day	Not specified	Immediately post expansion
Wendling et al. (2005) [15]	Prospective	9 years 8 months	Not specified	25 (15/10)	1 turn/day	Mean of 9.7 months	At 5 months retention

**Table 4:** The change in skeletal and dentoalveolar parameters between pre- and post-treatment records as measured on lateral cephalograms, extracted from the studies identified through our systematic review. T1: pre-treatment records; T2: immediately post-expansion records; T3: post-retention records. The data are presented as mean ± standard deviation, except the ones with additional notes. SE: standard error; N: nasion; ANS: anterior nasal spine; mm: millimeters; Me: menton; Ba: basion; Pt: Pt point; Gn: gnathion; S: sella; PP: palatal plane; PNS: posterior nasal spine; U6: upper first molar; MP: mandibular plane; FH: Frankfort Horizontal; Go: gonion; L6: lower first molar.

Parameter	Article	T1	T2	T3	T2-T1 Difference	T3-T1 Difference
N-ANS (mm)	De Rossi et al. (2010) [8]	45.96 ± 2.92		46.52 ± 3.76		0.56 ± 1.41
	Doruk et al. (2004) [14]	55.32 ± 3.25	56.62 ± 3.44	56.32 ± 3.36	1.30 ± 2.39	1.00 ± 1.75
	Mara Galon et al. (2003) [11]	47.00 ± 3.718		48.316 ± 3.882		
	Pinto et al. (2012) [7]	45.6777 ± 2.8383		46.0577 ± 3.0975		0.3800 ± 1.2889
N-Me (mm)	De Rossi et al. (2010) [8]	106.72 ± 5.07		107.76 ± 5.24		1.04 ± 1.83
	Mara Galon et al. (2003) [11]	107.860 ± 6.708		109.326 ± 6.179		
	Pinto et al. (2012) [7]	108.5315 ± 4.8511		109.1273 ± 5.1055		0.5958 ± 1.8994
ANS-Me (mm)	Asanza et al. (1997) [6]	69.88 (range: 63.9 to 79.6)				0.05 (range: −1.70 to 2.00)
	De Rossi et al. (2010) [8]	63.08 ± 4.06		63.72 ± 3.92		0.64 ± 1.97
	Doruk et al. (2004) [14]	68.56 ± 4.63	71.15 ± 5.56	70.65 ± 5.37	2.59 ± 3.10	2.09 ± 2.22
	Mara Galon et al. (2003) [11]	62.975 ± 4.261		63.943 ± 3.300		
	Pinto et al. (2012) [7]	62.8527 ± 3.7512		63.0704 ± 3.9912		0.2177 ± 1.5093
	Wendling et al. (2004) [15]	64.7 ± 5.2				0.2 ± 1.4
PP-Po-Or (°)	Pinto et al. (2012) [7]	4.1850 ± 2.7565		3.8612 ±2.7186		−0.3238 ± 1.4011
Condylion to gonion (mm)	Wendling et al. (2004) [15]	50.1 ± 3.8				0.7 ± 1.8
Gonion to pogonion (mm)	Wendling et al. (2004) [15]	71.6 ± 3.6				0.9 ± 1.6
Mandibular length (Co-Gn) (mm)	Wendling et al. (2004) [15]	108.5 ± 5.6				1.5 ± 1.2
Total facial height (NaBa-XiPm) (°)	Conroy-Piskai et al. (2016) [13]	65.92 ± 2.27		65.84 ± 2.49		−0.07
Lower facial height (ANS-Xi-Pm) (°)	Conroy-Piskai et al. (2016) [13]	48.28 ± 3.13		48.67 ± 3.16		0.38
Bjork sum (degrees)	Hasan et al. (2019) [10]	398.85 ± 5.09		399.51 ± 3.96		
Y-axis (SN-SGn) (degrees)	Hasan et al. (2019) [10]	70.84 ± 4.6		71.17 ± 3.5		
PFH/AFH (%)	Hasan et al. (2019) [10]	61.28 ± 3.4		60.93 ± 2.9		
UFH/LFH (%)	Hasan et al. (2019) [10]	79.9 ± 5.1		79.3 ± 4.9		
SN-PP angle (°)	Asanza et al. (1997) [6]	8.07 (range: 0 to 12.4)				1.25 (range: 0.20 to 2.80)
	De Rossi et al. (2010) [8]	7.88 ± 3.44		7.40 ± 3.31		−0.48 ± 1.75
	Doruk et al. (2004) [14]	9.65 ± 2.66	8.94 ± 2.34	9.21 ± 2.38	−0.71 ± 1.87	−0.44 ± 1.66
	Mara Galon et al. (2003) [11]	8.063 ± 3.462		8.636 ± 3.281		
	Pinto et al. (2012) [7]	6.8831 ± 2.7199		6.7908 ± 2.8008		−0.0923 ± 1.7113
	Sarver et al. (1989) [5]					0.50 ± 0.30 (SE)
	Wendling et al. (2004) [15]	6.8 ± 4.2				0.5 ± 1.2
SN-PNS (mm)	Asanza et al. (1997) [6]	44.0 (range: 41.6 to 49.4)				0.50 (range: −1.20 to 1.50)
	Sarver et al. (1989) [5]					0.35 ± 0.18 (SE)
SN-ANS (mm)	Asanza et al. (1997) [6]	51.8 (range: 46.3 to 58.5)				1.50 (range: 0.40 to 4.20)
	Sarver et al. (1989) [5]					1.25 ± 0.19 (SE)
SN–occlusal plane angle (°)	Akkaya et al. (1999) [12]	25.94 ± 1.19	26.85 ± 1.17	25.89 ± 0.97		
	De Rossi et al. (2010) [8]	19.24 ± 3.97		19.00 ± 4.67		−0.24 ± 2.87
	Pinto et al. (2012) [7]	20.0415 ± 4.0931		20.1308 ± 4.3207		0.0893 ± 1.5746
	Wendling et al. (2004) [15]	20.8 ± 5.0				−0.3 ± 3.2
U6-PP (mm)	Asanza et al. (1997) [6]	22.23 (range: 17.0–26.0)				−0.90 (range: −2.50 to 0.75)
	Conroy-Piskai et al. (2016) [13]	19.62 ± 1.79		19.29 ± 2.07		−0.33
L6-MP (mm)	Conroy-Piskai et al. (2016) [13]	26.79 ± 2.27		27.66 ± 2.62		0.87
SN-MP angle (°)	Akkaya et al. (1999) [12]	39.40 ± 0.84	41.33 ± 0.99	40.69 ± 0.85		
	Doruk et al. (2004) [14]	39.09 ± 7.26	40.94 ± 7.17	40.50 ± 7.18	1.85 ± 1.37	1.41 ± 1.03
	Pinto et al. (2012) [7]	39.1738 ± 5.2802		38.8958 ± 5.2642		−0.2781 ± 1.7458
	Sarver et al. (1989) [5]					0.75 ± 0.39
	Wendling et al. (2004) [15]	35.3 ± 6.1				0.2 ± 1.0
MP-FH angle (FMA) (°)	Conroy-Piskai et al. (2016) [13]	29.26 ± 2.32		29.55 ± 2.60		0.29
	Pinto et al. (2012) [7]	26.0965 ± 4.4902		26.2550 ± 4.5948		0.1585 ± 1.4388
PP-GoGn angle (°)	De Rossi et al. (2010) [8]	29.40 ± 4.17		29.92 ± 3.35		0.52 ± 2.16
	Mara Galon et al. (2003) [11]	27.642 ± 3.686		28.983 ± 4.282		
	Pinto et al. (2012) [7]	30.1196 ± 4.0914		30.0346 ± 4.1125		−0.0850 ± 2.1920
SN-GoGn angle (°)	De Rossi et al. (2010) [8]	37.28 ± 5.31		37.36 ± 4.79		0.08 ± 1.60
	Mara Galon et al. (2003) [11]	35.706 ± 3.810		37.613 ± 4.302		
	Pinto et al. (2012) [7]	37.0612 ± 5.2509		36.8019 ± 5.2126		−0.2593 ± 1.7869
SN-Gn angle (°)	De Rossi et al. (2010) [8]	68.88 ± 4.52		68.92 ± 4.61		0.04 ± 1.05
	Mara Galon et al. (2003) [11]	67.154 ± 3.581		68.986 ± 3.782		
	Pinto et al. (2012) [7]	69.0262 ± 4.5551		68.7873 ± 4.5124		−0.2389 ± 1.1776
Facial axis (NaBa-PtGn) (°)	Conroy-Piskai et al. (2016) [13]	84.07 ± 2.22		83.64 ± 2.38		−0.43
	De Rossi et al. (2010) [8]	85.16 ± 3.28		85.04 ± 4.01		−0.12 ± 2.12

## Data Availability

All data generated or analyzed during this study are included in this published article.
